# Supplementary material for: Religious and cultural perspectives on assisted reproductive technology in Ghana: A comparative analysis of traditionalist, islamic, and christian beliefs
Source: PLOS Glob Public Health. 2025 Sep 25;5(9):e0005240. doi: 10.1371/journal.pgph.0005240 (PMC12463212; doi:10.1371/journal.pgph.0005240)
Supplement: S1 Text — (DOCX) [file pgph.0005240.s001.docx]

**S1 Text: Study Instrument**

**Title:** Religious Perspectives on Assisted Reproductive Technology (ART)

**Objective:** To gather insights from religious leaders on their beliefs, experiences, and interpretations of ART within their faith traditions.

**Interview Duration:** 45–60 minutes

**Language:** English (with an option to use local dialects if needed)

**Section 1**

**Religious Teachings on Childbearing:**

- - How does your faith view the importance of childbearing and family?
  - Are there specific teachings or scriptures that emphasise reproduction?

**Spiritual Significance of Parenthood:**

- - In what ways do religious beliefs influence attitudes toward conception and infertility?
  - How does your faith address challenges related to infertility?

**Section 2**

**Awareness & Understanding of ART:**

- - How familiar are you with ART methods such as IVF (In Vitro Fertilisation), surrogacy, and sperm or egg donation?
  - Does your faith community generally support or oppose these procedures? Why?

**Ethical & Moral Considerations:**

- - What ethical concerns does your religion raise about ART?
  - Are there specific ART practices that align with or conflict with religious values?

**Religious Approval or Disapproval:**

- - Does your faith tradition officially permit or discourage ART?
  - What guidance do religious texts provide regarding artificial conception methods?

**Surrogacy and Third-Party Reproduction:**

- - How does your faith view the use of surrogacy or third-party donors (sperm/egg donation)?
  - Are there concerns about lineage, inheritance, or moral integrity?

**Role of Religious Leaders in Decision-Making:**

- How do religious leaders counsel couples facing infertility or considering ART?
- Have you personally guided any members on ART-related decisions?

**Faith-Based Alternatives or Rituals:**

- Are there religious ceremonies, prayers, or healing practices used to address infertility?
- Do faith communities encourage spiritual approaches over medical solutions?

**Community Perceptions & Acceptance:**

- How do congregations react to couples using ART?
- Are there cases of stigma or support within your faith community for children born through the use of ART?

**Section 3**

**Religious Adaptation to Medical Advances:**

- How do you see religious perspectives evolving as ART becomes more common?
- Should religious teachings be revisited in light of advancements in reproductive technology?
- Is there any additional insight you would like to share regarding faith and ART?
- What advice would you give to policymakers or health professionals when discussing ART within religious settings?

**Focus Group Discussion (FGD) Guide for Congregants**

**Title:** Religious Perspectives on Assisted Reproductive Technology (ART)

**Objective:** To explore congregants' views on infertility and ART within their religious beliefs and community settings.

**Participants:** Members of various religious groups (Christian, Muslim, Traditional)

**Discussion Duration:** 60–90 minutes

**Language:** English and local Twi as needed

**Section 1**

**Perceptions of Infertility in Religion**

- How does your faith interpret infertility? Probe for detailed answers
- What are the common beliefs about its causes and effects? Probe

**Religious Responses to Infertility**

- Does your religion provide spiritual or faith-based solutions for infertility?
- Are there rituals, prayers, or traditional practices used for fertility issues? Probe

**Section 2**

**Acceptance of ART Practices and Beliefs in Faith Communities**

- How do religious teachings view ART as a treatment option?
- What are the general attitudes of religious leaders and congregants toward ART?
- How do you feel about gamete donation, surrogacy, and embryo cryopreservation?

**Ethical & Moral Considerations**

- Are there specific religious principles that support or oppose ART?
- Do faith communities encourage natural conception over medical intervention?

**Section 3**

**Perception of ART & Children Born Through It**

- How do religious groups view children conceived through ART?
- Are there cultural or spiritual concerns regarding their status in the faith community?
- What role should religious institutions play in informing and supporting infertile couples?

**Personal Perspectives & Open Discussion**

- Should infertile couples within your faith consider ART? Why or why not?
- What would you like religious leaders and policymakers to understand about ART?
- Is there anything else you would like to share about your faith and ART?
