# Supplementary material for: Religious and cultural perspectives on assisted reproductive technology in Ghana: A comparative analysis of traditionalist, islamic, and christian beliefs
Source: PLOS Glob Public Health. 2025 Sep 25;5(9):e0005240. doi: 10.1371/journal.pgph.0005240 (PMC12463212; doi:10.1371/journal.pgph.0005240)
Supplement: Attachment — Submitted filename: PLOS-Religions-Review-Aug24.docx [file pgph.0005240.s002.docx]

Dear Author

First of all I would like to congratulate you for this careful and interesting work. It is a very "Ghanaian" look at aspects of infertility and assisted reproduction, but at the same time we can extrapolate some of these feelings and testimonies as universal.

Acronyms

The paper is well written, only one observation is related to the acronyms, which should be next to the first reference, in parentheses. This does not occur, for example, in Design, where semi-stuctured in depth interviews and focus group discussions are not followed by IDI and FGDs. Next, when reading Sampling and data collection, we have to go back to understand the acronyms.

In the fourth line of discussion, the acronym CAM is used for complementary medicine. However, this reference is not repeated, and is probably dispensable.

Sampling and data collection

The author states that Christian individuals (Orthodox, Protestant and Catholic) were heard, as well as Muslims and members of the Traditional African religion, religious figures and practising members of the communities.

Later Methodists, Anglicans, and Presbyterians are cited as Orthodox, others distinct as Pentecostals, Assemblies of God Church, and Light House Chapel. This nomination as Orthodox seems to me not adequate. Perhaps better consider them all as Christians, yes, but Protestants. In fact the last three in my country (Brazil) probably being more radical.

Traditional- was the group that caught my attention, for an increasingly radical conduct. Would they be older or with a lower degree of formal education?

Religious leaders tended to position themselves more vehemently against the procedures of gamete donation, cryopreservation and surrogacy. It is interesting their citing of spiritual consequences, while 2 practicing Methodists would consider either as adultery (man), or the woman leaving the church to undergo treatment.

The Traditional group again intrigued me by attributing to assisted reproduction the desire of man to compare himself to God, going against God's will, adultery etc. Sometimes it seems to me more like a position of other religions in their radical branches. Wasn't traditional herbal medicine associated with amulets, offerings, or the like?

And, was there no condition of religious syncretism reported in these testimonies of practitioners? I expected to find a traditionalist mention of various entities of gods, not a single reference.

Finally, the testimonies collected among the practitioners demonstrate a culturally evolving Ghanaian society, where concepts related to family foundation can be better defined.

Regards.
