# Supplementary material for: Religious and cultural perspectives on assisted reproductive technology in Ghana: A comparative analysis of traditionalist, islamic, and christian beliefs
Source: PLOS Glob Public Health. 2025 Sep 25;5(9):e0005240. doi: 10.1371/journal.pgph.0005240 (PMC12463212; doi:10.1371/journal.pgph.0005240)
Supplement: Attachment — Submitted filename: Response to reviewers comments.docx [file pgph.0005240.s005.docx]

September 23, 2024

Dear Editor

**Response to Reviewers' comments**

Thank you for sending the reviewers’ comments on the manuscript **"Exploring the Intersection of Religion and the Acceptance of Assisted Reproductive Technology Practices: An Examination of Selected Religious Groups in Ghana"** to me for the opportunity to address them for your consideration. I have addressed all the comments and provided some responses and clarifications where necessary as per the attached. I have also attached a track-changed and untracked version for your review. I believe the manuscript has improved vastly and look forward to your kind consideration for acceptance and publication.

Thank you.

Kwadwo Asante-Afari

(Correspondent)

Reviewer(s)' Comments to Author:

**Acronyms**

1. Comment: In-depth interviews and Focus Group Discussions were not followed by acronyms.  When reading Sampling and data collection, we have to go back to understand the acronyms.

**Response: Acronyms for In-Depth Interviews (IDIs) and Focus Group Discussions (FGDs) are defined upon first use and used consistently throughout the study. See pages 5-7 for details.**

2. Comment: The author states that Christian individuals (Orthodox, Protestant and Catholic) were heard, as well as Muslims and members of the Traditional African religion, religious figures and practising members of the communities.
   Later Methodists, Anglicans, and Presbyterians are cited as Orthodox, others distinct as Pentecostals, Assemblies of God Church, and Light House Chapel. This nomination as Orthodox seems to me not adequate. Perhaps better to consider them all as Christians, yes, but Protestants. In fact, the last three in my country (Brazil) probably being more radical.

**Response: Referring to Methodists, Anglicans, and Presbyterians as Orthodox has been changed to Protestants as suggested by the reviewer. Please see paragraph 1 of page 6 for details**

3. Comment: In the fourth line of discussion, the acronym CAM is used for complementary medicine. However, this reference is not repeated and is probably dispensable.

**Response: The use of the CAM acronym has been discontinued because of its misplaced application.**

Comments: Traditional- was the group that caught my attention, for an increasingly radical conduct. Would they be older or with a lower degree of formal education?

**Response: Most of the respondents who belonged to the Traditional African religion were old (40+ years) and had a lower degree of formal education. See the first paragraph under ‘Respondent background characteristics’ on page 10 for further details**

**Comment**

The Traditional group again intrigued me by attributing to assisted reproduction the desire of man to compare himself to God, going against God's will, adultery etc. Sometimes it seems to me more like a position of other religions in their radical branches. Wasn't traditional herbal medicine associated with amulets, offerings, or the like?

**Response: Yes traditional herbal medicine was associated with amulets, offerings, or the like but also related to the taking of herbal medication, concoctions, the wearing of artefacts and many more. Please see pages 11 and 18 (first paragraph) under discussion for information on this comment.**

Comment

And, was there no condition of religious syncretism reported in these testimonies of practitioners? I expected to find a traditionalist mention of various entities of gods, but not a single reference

**Response: there was a condition of religious syncretism reported in these testimonies of practitioners. For example, on page 18, participants from both Christian and Islamic backgrounds reported combining traditional and orthodox medicine with prayer (religion and medicine) to address infertility and this is a clear example of religious syncretism. This has been explained in the first paragraph of page 18 (Discussions)**

Response **References have been duly revised and crosschecked to ensure that all references cited in intext have been addressed in the main reference column**. Please see the reference section for further details
