# Supplementary material for: Religious and cultural perspectives on assisted reproductive technology in Ghana: A comparative analysis of traditionalist, islamic, and christian beliefs
Source: PLOS Glob Public Health. 2025 Sep 25;5(9):e0005240. doi: 10.1371/journal.pgph.0005240 (PMC12463212; doi:10.1371/journal.pgph.0005240)
Supplement: Attachment — Submitted filename: Review Report.docx [file pgph.0005240.s004.docx]

**Review Report**

Title of the Paper **“Exploring the Intersection of Religion and the Acceptance of Assisted Reproductive Technology Practices: An Examination of Selected Religious Groups in Ghana".**

The paper explores the role of religious beliefs in shaping attitudes toward Assisted Reproductive Technologies (ART) in Ghana, with a focus on Traditionalist, Islamic, and Christian perspectives. It argues that religion and culture strongly influence the acceptance or rejection of ART practices, particularly those involving third-party reproductive resources.

**Strengths**

1. The paper presents a rich, descriptive analysis of religious perspectives on Assisted Reproductive Technologies (ART), focusing on Traditionalist, Islamic, and Christian views in Ghana.
2. The integration of qualitative evidence and references to prior studies is commendable, enhancing the depth of cultural and religious context.
3. The discussion of syncretism - how individuals blend religious, traditional, and medical practices – is an important contribution to understanding ART in sub-Saharan Africa.

**Weaknesses and Recommendations**

1. Lack of Nuance in Islamic Perspectives: While Islamic views on ART are discussed, the paper overlooks key theological differences between Sunni and Shia interpretations. For example, Shia scholars tend to be more permissive regarding ART practices such as donor gametes compared to Sunni scholars, who often adhere strictly to prohibitions on third-party involvement. This omission weakens the paper’s comprehensive coverage of religious diversity in Islam.

Furthermore, the paper does not cite recent works specifically addressing Shia perspectives on ART. Given the rich body of literature on this subject, including studies exploring Shia theological reasoning and its implications for reproductive ethics, incorporating such perspectives would provide a more comprehensive analysis of Islamic views on ART.

**Recommendation:** Incorporate a comparative analysis of Sunni and Shia perspectives to provide a nuanced understanding of Islamic views on ART. Cite works on Shia interpretations.

2. **Absence of Critical Engagement with ART Ethics:** The ethical implications of ART practices, such as donor gametes, surrogacy, and cryopreservation, are mentioned but not critically analyzed. For instance, the claim that donor gametes could lead to incest is stated without sufficient evidence or theoretical critique.

**Recommendation:** Provide a more robust ethical analysis, drawing on both religious and secular bioethical frameworks. This could include discussing the rights of children born through ART or the social implications of third-party involvement in reproduction.

3. **Over-Reliance on Descriptive Evidence:** While the descriptive accounts are valuable, the paper lacks a strong analytical framework to tie the findings to broader theoretical debates on religion and medical technology. The central argument could benefit from a stronger synthesis of the key findings. For instance, the paper could explicitly compare and contrast the shared and divergent viewpoints among the three groups.
4. **Conclusion:** The conclusion effectively summarizes the findings but could be strengthened by linking them to broader global debates on ART and religion.
5. Words like Muslims instead of Moslems should be used.
6. The title, "Exploring the Intersection of Religion and the Acceptance of Assisted Reproductive Technology Practices: An Examination of Selected Religious Groups in Ghana", is generally descriptive and provides a clear sense of the study's focus. However, it could be refined for greater precision and to better reflect the content and objectives of the paper.

**Recommendation:** To make the title more precise and reflective of the paper's contributions, consider revising it to: "Religious and Cultural Perspectives on Assisted Reproductive Technology in Ghana: A Comparative Analysis of Traditionalist, Islamic, and Christian Beliefs"

**Final Evaluation**

This paper makes a valuable contribution to understanding the intersection of religion, culture, and ART in Ghana, but it requires a more nuanced discussion of Islamic sectarian differences, a comparison among the three religions and a deeper exploration of ethical and cultural dimensions. With these improvements, the study could significantly advance the field.
