# Supplementary material for: Religious and cultural perspectives on assisted reproductive technology in Ghana: A comparative analysis of traditionalist, islamic, and christian beliefs
Source: PLOS Glob Public Health. 2025 Sep 25;5(9):e0005240. doi: 10.1371/journal.pgph.0005240 (PMC12463212; doi:10.1371/journal.pgph.0005240)
Supplement: Attachment — Submitted filename: Response_to_reviewers_comments_auresp_2.docx [file pgph.0005240.s006.docx]

February 26, 2025

Dear Editor

**Response to Editor’s comments**

Thank you for sending the reviewers’ comments on the manuscript **"Exploring the Intersection of Religion and the Acceptance of Assisted Reproductive Technology Practices: An Examination of Selected Religious Groups in Ghana"** to me for the opportunity to address them for your consideration. I have addressed all the comments and provided some responses and clarifications where necessary as per the attached. I have also attached a track-changed and untracked version for your review. I believe the manuscript has improved vastly and look forward to your kind consideration for acceptance and publication.

Thank you.

Kwadwo Asante-Afari

(Correspondent)

Reviewer(s)' Comments to Author:

Weaknesses and Recommendations

1. **Comment:** Lack of Nuance in Islamic Perspectives: While Islamic views on ART are discussed, the paper overlooks key theological differences between Sunni and Shia interpretations. For example, Shia scholars tend to be more permissive regarding ART practices such as donor gametes compared to Sunni scholars, who often adhere strictly to prohibitions on third-party involvement. This omission weakens the paper’s comprehensive coverage of religious diversity in Islam.

Furthermore, the paper does not cite recent works specifically addressing Shia perspectives on ART. Given the rich body of literature on this subject, including studies exploring Shia theological reasoning and its implications for reproductive ethics, incorporating such perspectives would provide a more comprehensive analysis of Islamic views on ART.

Recommendation: Incorporate a comparative analysis of Sunni and Shia perspectives to provide a nuanced understanding of Islamic views on ART. Cite works on Shia interpretations.

**Response: 1**

**The paper incorporates a comparative analysis of the Suni and Shia perspectives on ART practices. Further, current references have been cited in both Suni and Shia perspectives. See pages 19, 20, 22, and 23 for details.**

2. **Comment:** Absence of Critical Engagement with ART Ethics: The ethical implications of ART practices, such as donor gametes, surrogacy, and cryopreservation, are mentioned but not critically analyzed. For instance, the claim that donor gametes could lead to incest is stated without sufficient evidence or theoretical critique.

Recommendation: Provide a more robust ethical analysis, drawing on both religious and secular bioethical frameworks. This could include discussing the rights of children born through ART or the social implications of third-party involvement in reproduction.

**Response 2**

**The ethical analysis of the rights of children born through ART has been discussed on pages 24-25 for detailed analysis.**

3. **Comment:** Over-Reliance on Descriptive Evidence: While the descriptive accounts are valuable, the paper lacks a strong analytical framework to tie the findings to broader theoretical debates on religion and medical technology. The central argument could benefit from a stronger synthesis of the key findings. For instance, the paper could explicitly compare and contrast the shared and divergent viewpoints among the three groups.

**Response 3**

**Under each of the key findings, shared and divergent viewpoints among the three groups have been discussed. Literature from other studies supports these arguments. Please see the discussion section of the paper for evidence.**

4. Conclusion: The conclusion effectively summarizes the findings but could be strengthened by linking them to broader global debates on ART and religion.

**Response 4**

**The conclusion has been comprehensively discussed and linked to the current global debate on Art and region. Please refer to pages 24-25 for the details.**

5. Words like Muslims instead of Moslems should be used.

**Response 5**

**Moslems have been duly replaced with Muslims in the transcript (See the entire paper for the changes).**

6. The title, "Exploring the Intersection of Religion and the Acceptance of Assisted Reproductive Technology Practices: An Examination of Selected Religious Groups in Ghana", is generally descriptive and provides a clear sense of the study's focus. However, it could be refined for greater precision and to better reflect the content and objectives of the paper.

Recommendation: To make the title more precise and reflective of the paper's contributions, consider revising it to: "Religious and Cultural Perspectives on Assisted Reproductive Technology in Ghana: A Comparative Analysis of Traditionalist, Islamic, and Christian Beliefs"

**Response 6.**

**The title has been revised to reflect the suggestion made by the reviewers. The new title reads**

**"Religious and Cultural Perspectives on Assisted Reproductive Technology in Ghana: A Comparative Analysis of Traditionalist, Islamic, and Christian Beliefs" see the title page for evidence**
