# Supplementary material for: Religious and cultural perspectives on assisted reproductive technology in Ghana: A comparative analysis of traditionalist, islamic, and christian beliefs
Source: PLOS Glob Public Health. 2025 Sep 25;5(9):e0005240. doi: 10.1371/journal.pgph.0005240 (PMC12463212; doi:10.1371/journal.pgph.0005240)
Supplement: Attachment — Submitted filename: Response to reviewers comments (2).docx [file pgph.0005240.s007.docx]

May 21, 2025

Dear Editor

**Response to Editor’s comments**

Thank you for sending the reviewers’ comments on the manuscript **"Exploring the Intersection of Religion and the Acceptance of Assisted Reproductive Technology Practices: An Examination of Selected Religious Groups in Ghana"** to me for the opportunity to address them for your consideration. I have addressed all the comments and provided some responses and clarifications where necessary as per the attached. I have also attached a track-changed and untracked version for your review. I believe the manuscript has improved vastly and look forward to your kind consideration for acceptance and publication.

Thank you.

Kwadwo Asante-Afari

(Correspondent)

Reviewer(s)' Comments to Author:

**Comment 1:** In the introduction section, the author(s) indicate that “In Ghana, approximately 15% of women of childbearing age are estimated to experience infertility (Donkor & Sandall, 2009).”That was in 2009; however, what is the current data saying about the women of childbearing age estimated to experience infertility in Ghana?

**Response: A current data on infertility in Ghana has been inserted into the introductory sentence. See page 1 for details**

**Comment 2:** Kindly provide citations for the facts presented in the section titled “setting.”

**Response: The current data source has been indicated under the sub-title ‘setting’ The Ghana Living Standard Survey is conducted every 20years. The 8th edition was started in 2024 but the results are not yet out. As a result the 2014 edition is being used. Refer to page 5 for details**

**Comment 3:** The authors must also explain how they ensured a fair representation of the study's participants, even though they used purposive sampling.

**Response:** **The study ensured fair representation by including participants from Islamic, Christian, and Traditional religious backgrounds, promoting diverse perspectives on ART. Both males and females were involved to minimise gender biases. Additionally, a mix of age groups, statuses, and experiences within the religious community ensured a broader and more representative sample. See page 8 for more explanations**

Comment 4: The author(s) indicate that the interviews were conducted in English and Twi. Can they indicate the number of interviews carried out in English and those carried out in Twi?

**Response: All IDIs (6) were conducted in English, while a combination of English and Twi was used in the FGDs involving 24 people (2 male groups and three female groups) for clarity and emphasis. This approach also accommodated six individuals who were unable to express themselves in English, allowing them to communicate in their local dialect. Please see pages 5 and 6 for details.**

**Comment 5:** Also, in what language was the focus group discussion carried out?

**Response: A combination of English and Twi was used for the FGDs. See pages 5 and 6.**

**Comment 6**: Were the participants in the focus group selected from the initial sample? If so, could you provide information about their background, including whether they were Islamic, Christian, or traditional? Only stating the numbers is not enough.

**Response: The FGDs participants were a mix of people from the three religious groups. This is indicated on page 7 of the manuscript**

**Comment 7:** Who translated the interview in Twi?

**Response: The author who is the principal investigator and well versed (speak fluently and write) in Twi translated the interview in Twi. This stated on page 7 of the manuscript**

**Comment 8:** The author(s) should clearly indicate the implications of their study.

**Response: The implication of the study has been added to the manuscript. See page 26 for details.**

Comment 9: The author(s) should add the interview questions as an appendix.

**Response:**

**The interview questions have been added to the manuscript as appendix. See pages 32-34**
