# Supplementary material for: Religious and cultural perspectives on assisted reproductive technology in Ghana: A comparative analysis of traditionalist, islamic, and christian beliefs
Source: PLOS Glob Public Health. 2025 Sep 25;5(9):e0005240. doi: 10.1371/journal.pgph.0005240 (PMC12463212; doi:10.1371/journal.pgph.0005240)
Supplement: Attachment — Submitted filename: Response_to_reviewers_comments_auresp_4.docx [file pgph.0005240.s008.docx]

July 20, 2025

Dear Editor

**Response to Editor’s comments**

Thank you for sending the reviewers’ comments on the manuscript **"Exploring the Intersection of Religion and the Acceptance of Assisted Reproductive Technology Practices: An Examination of Selected Religious Groups in Ghana"** to me for the opportunity to address them for your consideration. I have addressed all the comments and provided some responses and clarifications where necessary as per the attached. I have also attached a track-changed and untracked version for your review. I believe the manuscript has improved vastly and look forward to your kind consideration for acceptance and publication.

Thank you.

Kwadwo Asante-Afari

(Correspondent)

Reviewer(s)' Comments to Author:

**Comment 1:** Kindly follow COREQ checklist in your manuscript writing

**Response: The author has checked and followed COREQ checklist	in coming out with the revised manuscript**

**Comment 2:** Reflexivity statement is required

**Response:** The author has incorporated a reflexivity statement within	the manuscript to address positionality and research perspective. For full	details, refer to page 29.

**Comment 3:** Authors are required to draw upon principles of human dignity in understanding the themes that emerged from the study. Dignitas infinita is a useful document to refer to

**Response: The author has read and cited Dignitas Infita in portions	of the work. Please refer to pages 22 and 23 of the manuscript**

**Comment 4:** Conceptual framework diagram is required for better understanding

**Response:** **Although an ideal conceptual framework specific to the	study’s focus was not readily available in existing literature, the	author identified a theoretical framework that aligns meaningfully	with the research objectives. This framework offers a solid analytical	foundation and guides the interpretation of findings, particularly in	relation to the intersection of religious and cultural perspectives on	ART in Ghana. For a comprehensive explanation of the selected	theoretical framework, refer to page 4 of the manuscript.**
Comment 5: Authors are requested to bring in the social construct of gender and patriarchy in understanding the statements mentioned by participants.

**Response: The author has thoughtfully integrated the concepts of	gender as a social construct and patriarchy into the discussion	section, with appropriate scholarly references to support the analysis.	See pages 21-23**

References:

**All other references cited in support of the supplementary information have been duly incorporated into the main reference list.**
