# Supplementary material for: Religious and cultural perspectives on assisted reproductive technology in Ghana: A comparative analysis of traditionalist, islamic, and christian beliefs
Source: PLOS Glob Public Health. 2025 Sep 25;5(9):e0005240. doi: 10.1371/journal.pgph.0005240 (PMC12463212; doi:10.1371/journal.pgph.0005240)
Supplement: Attachment — Submitted filename: Response_to_reviewers_comments_auresp_5.docx [file pgph.0005240.s009.docx]

August 25, 2025

Dear Editor

**Response to Editor’s comments**

Thank you for sending the reviewers’ comments on the manuscript **"Exploring the Intersection of Religion and the Acceptance of Assisted Reproductive Technology Practices: An Examination of Selected Religious Groups in Ghana"** to me for the opportunity to address them for your consideration. I have addressed all the comments and provided some responses and clarifications where necessary as per the attached. I have also attached a track-changed and untracked version for your review. I believe the manuscript has improved vastly and look forward to your kind consideration for acceptance and publication.

Thank you.

Kwadwo Asante-Afari

(Correspondent)

**Statistical and methodological clarification**

**Comment 1**: Clarification of participant distribution: While the demographic breakdown in Table 2 is helpful, the qualitative sample of 30 participants could benefit from a clearer explanation of the rationale behind its size and how thematic saturation was determined. A brief mention of the principles guiding saturation in qualitative research (e.g., Guest et al., 2006) would strengthen methodological transparency.

**Response 1: A brief mention has been made of the principle guiding	saturation in the study. Please see page 7**

**Comment 2:** Duration and timing: The recruitment period (March–August 2017) should be briefly addressed in the discussion—consider adding a sentence explaining whether these positions have changed in recent years and how the passage of time affects current applicability**.**

**Response 2: The position on the findings from the study has not	changed although data for the study was collected in 2017. Further	explanation is indicated on page 29 under the conclusion.**

**Comment 3:** Language and style adjustments in English

- **While the manuscript is generally well written, I recommend a final professional proofreading to improve clarity. Here are some sample corrections and stylistic suggestions:**

**Summary:**

- **Source: "...while respecting people's religious beliefs and values."**
- **Suggested: "...while respecting people's religious beliefs and values."**

**Page 13:**

**Source: "...this act is outright prostitution..."**

**Suggested: "...this act is akin to prostitution..."**

**(To maintain an academic tone)**

**Page 17:**

**Source: "Why freeze your eggs or sperm?"**

**Suggested: "What is the purpose of freezing a person's eggs or sperm?"**

**(For clarity and professionalism.)**

**Response: 3** The whole paper has been proof read and the necessary	corrections done. Check the entire document**.** Check pages 13 and 17 for	details

**Comment 4: General: The use of terms such as "dirty," "illegitimate," and "abomination" appears in direct quotes from participants. Consider marking them as culturally or contextually situated in the analysis to avoid unintentional offense or misinterpretation.**

**Response: 4** Culturally accepted terms have replaced terms such	abomination, many others. Check page 17 for more details

**Comment 5: Commentary on Religious Frameworks - Including Bokek's Contribution**

**Response:5** The combination of the God Centric and ecological models	underpin the present study. The theoretical triangulation was	influenced by the work of Bokek et al 2022, which combined the	ecological model and context-informed perspective. Excerpts have	been integrated into the study. See pages 4&5 for detail.

**Comment 6: The manuscript addresses religious perspectives well, but the excellent contributions of Ya'arit Bokek-Cohen and Tarabiyeh (2021, 2022) are only briefly mentioned. Given the direct relevance of their findings to the Islamic and Christian ethics of antiretroviral therapy, I highly recommend the following:**

I. **Violating Religious Prohibitions to Preserve Family Harmony and Lineage among Sunni Muslims. Marriage & Family Review**
II. **What do Sunni Muslims think about religiously forbidden reproductive options?. Human fertility**
III. **Underground gamete donation in Sunni Muslim patients. Journal of religion and health**
IV. **The Ethical Standards of Sunni Muslim Physicians Regarding Fertility Technologies that are Religiously Forbidden**

**Response 6:** The suggested articles have read into detail and have been	helpful. They have been cited to support my findings. See the discussion	column for more details (pages 20,21,26 etc).

**Comment 7: Additional Integration: The studies of Bokek-Cohen et al. (2021, 2022) should be more thoroughly integrated into the discussion of Sunni Muslim views, especially regarding:**

I. **The secretive nature of gamete donation.**
II. **The tension between personal fertility desires and communal/religious prohibitions.**
III. **Gender dynamics in the deception and secrecy of partners in the use of antiretroviral therapy.**

**Response 7:** The studies of Bokek-Cohen et al. (2021, 2022) have been	thoroughly discussed and cited as references throughout the discussion.	See the discussion column for detail

**Comment 8: Comparative Insight: A few lines contrasting the Ghanaian religious context with other Muslim or Christian societies (e.g., Israel, Egypt, or Turkey) could enrich the discussion and global applicability.**

**Response 8:** This suggestion has been considered under the discussion.	Please refer to page 21 for details.

**Comment 9: This literature by Bokek-Cohen et al. may be referenced, as well as other literature if any.**

**Response 9:** Bokek-Cohen et al has been referenced under the Sunni	and Shia idscussions. See pages 20, 21, 22 and 23 for more details.

**Comment 10: Minor recommendations for future research**

**Future work should consider including:**

i. **Perspectives from young unmarried adults, who may represent changing generational perspectives.**
ii. **The legal and policy implications of antiretroviral treatment practices in Ghana, particularly around inheritance, adoption, and custody laws.**

**Response 10:** The minor recommendations are incorporated into the	conclusion on page 29 of the manuscript

**Comment 11: Add a brief note in the conclusion on how public health initiatives might engage religious leaders in designing more inclusive antiretroviral drug awareness campaigns.**

**Response** 11: This has been added to the conclusion on page 29 of the manuscript
